# The incidence and predictors of false-negative pancreatobiliary fluorescence in situ hybridization (PB-FISH) in biliary strictures: A prospective study

**DOI:** 10.1097/HC9.0000000000000751

**Published:** 2025-07-14

**Authors:** Manik Aggarwal, Gregory J. Gores, Eric J. Vargas, Andrew C. Storm, Ryan J. Law, Barham K. Abu Dayyeh, John A. Martin, Bret T. Petersen, John E. Eaton, Sumera I. Ilyas, Lewis R. Roberts, Vinay Chandrasekhara

**Affiliations:** 1 Division of Gastroenterology and Hepatology, Mayo Clinic, Rochester, Minnesota, USA; 2 Division of Gastroenterology and Hepatology, Cedars-Sinai Medical Center, Los Angeles, California, USA

**Keywords:** biliary, cancer, cholangiocarcinoma, stricture

## Abstract

**Background:**

Fluorescence in situ hybridization (FISH) is recommended as part of multimodality sampling in the evaluation of biliary strictures, but can be false negative in 40%–50% cases. The aim of this study was to comprehensively assess predictors of false-negative FISH in patients with biliary strictures.

**Methods:**

Patients undergoing tissue sampling for biliary strictures from October 20 to February 22 were prospectively enrolled (NCT04572711). Procedural factors such as sampling techniques, order, and utilization of combination sampling were recorded. Results of an optimized pancreatobiliary FISH panel with locus-specific probes for 1q21, 7p12, 8q24, and 9p21 were reported per guidelines. Statistical analyses were performed using BlueSky Statistics v.10.3.1, R package v8.81.

**Results:**

Of 327 patients, 93 (28.5%) were diagnosed with malignancy. A false-negative pancreatobiliary fluorescence in situ hybridization (PB-FISH) was reported in 40 (43.1%) patients. A history of primary sclerosing cholangitis was associated with lower odds of having a false-negative PB-FISH (aOR=0.38, 95% CI=0.16–0.82, *p*=0.02), whereas a hilar stricture was associated with a significantly higher false-negative PB-FISH in those with cholangiocarcinoma (aOR=3.2 [1.4–7.9], *p*=0.009). Dilation of biliary strictures prior to brushing was not associated with a lower rate of false-negative PB-FISH.

**Conclusions:**

In this prospective study, hilar strictures were more likely to have false-negative PB-FISH. Dilation of strictures did not affect PB-FISH performance. These results can help guide PB-FISH interpretation.

## Graphical abstract

**Figure:**
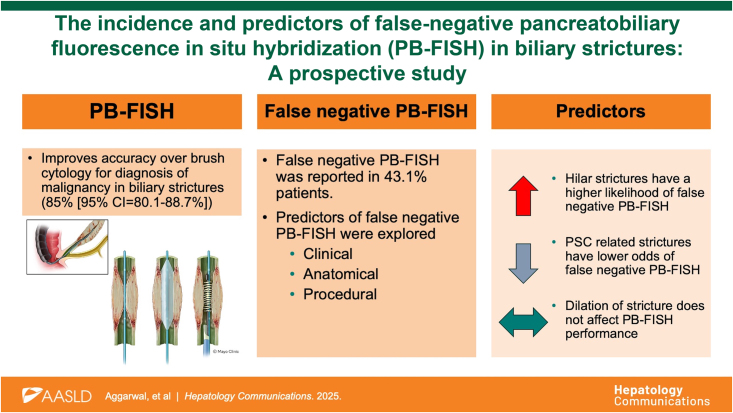

## INTRODUCTION

Early diagnosis of malignancy in biliary strictures can be challenging.^1^ Endoscopic retrograde cholangiopancreatography (ERCP) guided intraductal sampling of biliary strictures is the mainstay for safe and expedient diagnosis of pancreatobiliary (PB) malignancy in patients with biliary strictures.^2^^,^^3^ Traditional brush cytology (BC) is routinely used to establish a tissue-based diagnosis. However, BC is limited by poor sensitivity for malignancy and thus poses a clinical challenge.^2^^,^^4^

Recent guidelines recommend multimodality sampling of biliary strictures to enhance sensitivity for malignancy diagnosis.^2^ Fluorescence in situ hybridization (FISH) is an important component of multimodality sampling by virtue of significantly improved diagnostic sensitivity, in one study improving sensitivity to 42.9% for FISH combined with BC versus 20.1% for BC alone.^4^ An optimized pancreatobiliary fluorescence in situ hybridization (PB-FISH) assay developed specifically for PB malignancies has demonstrated further improved performance in comparison with the UroVysion assay that was designed for urothelial malignancies.^5^

Although significantly better than conventional cytology, PB-FISH may still be falsely negative in 30%–40% cases of malignancy. Inadequate cellularity of the specimen contributes to both false-negative BC and PB-FISH. Several factors, such as underlying primary sclerosing cholangitis (PSC), type of malignancy, location of strictures, and procedural factors (such as stricture dilation), have been described to affect BC performance, but factors affecting false-negative PB-FISH have not been studied.^3^^,^^6^^,^^7^ Evaluation of factors related to false-negative PB-FISH will improve test interpretation and guide further management. The aim of this study was to comprehensively assess the performance of PB-FISH and evaluate predictors of false-negative FISH in patients with biliary strictures, focusing on both anatomical and procedural factors.

## METHODS

### Patient selection

Adult patients ≥18 years of age undergoing ERCP for a biliary stricture were enrolled in the study at the time of ERCP between October 2020 and February 2022. This study was approved by the Mayo Clinic Institutional Board Review (IRB-20-004079) and enrolled in ClinicalTrials.gov (NCT04572711). This study was conducted in accordance with both the Declarations of Helsinki and Istanbul.

### ERCP procedure and data collection

PB strictures were sampled with biliary brushings either alone or in combination with dilation and/or transpapillary biopsy. Sampling techniques were based on the preference of the performing endoscopist. Procedural details, including the use of a biliary brush catheter, dilation and biopsy, and the order of each modality, were collected at the time of each procedure. Brushings were performed with the aim of sampling across the entire length of the stricture by performing 20 motions of the cytology brush. Each stricture location was sampled with one brush, and as per our clinical processing protocol, the sample was split for cytology and PB-FISH analysis. Select patients may have undergone biliary brushings in multiple distinct locations. After sampling, the brush catheter was flushed, and the tip was cut and placed into a vial of PreservCyt fixative (Hologic, Inc.) and then sent to the laboratory for further processing. Cell material was scraped from the brush into the solution, followed by equal division of the specimen for cytology and PB-FISH testing.

### Cytologic and histopathologic analysis

For routine cytology, 2 ThinPrep (Hologic, Inc.) slides were made from the cytology sample and then Pap-stained. A cytopathologist classified each case as nondiagnostic, negative, atypical, suspicious, or positive for malignancy based on the Papanicolaou Society of Cytopathology guidelines.^8^ BC was considered positive if the results were positive for malignancy.

### PB-FISH analysis

For PB-FISH testing, the FISH sample was centrifuged and treated with a fixative solution to obtain a cell pellet, and later the cells were manually dropped on a microscopic slide pretreated and hybridized with the refined pancreaticobiliary FISH probe set (1q21 [MCL1], 7p12 [EGFR], 8q24 [MYC], and 9p21 [CDKN2A]) (Abbott Molecular). FISH polysomy, defined as >3 copies of 2 or more probes (excluding tetrasomy), was classified as positive FISH. Other FISH observations were classified as negative. At least 100 epithelial cells with acceptable probe signal quality were required for specimen adequacy. This novel assay has demonstrated improved sensitivity when compared with both BC and the UroVysion assay and is the standard FISH assay at our institution.^5^

### Study definitions

Extrahepatic cholangiocarcinoma (CCA) was defined as a diagnosis of malignancy confirmed by either (1) positive cytology or histology or (2) established clinical criteria congruent with the current American Association for the Study of Liver Disease (AASLD) guidelines.^9^ In the absence of positive cytology or histology, any one of the following diagnostic criteria were considered definitive for perihilar CCA, (1) malignant appearing stricture with either a serum CA 19-9 >129 U/mL (in the absence of cholangitis or unstented obstructive jaundice), (2) PB-FISH polysomy, or (3) highly suspicious imaging findings (eg, a hilar mass with associated stricture, hypertrophy–atrophy complex, or associated vascular encasement).^10^ Any clinical diagnosis of CCA was made by the treating hepatologist, hepatobiliary surgeon, or medical oncologist. Serum CA 19-9 levels (lowest value within 30 days of the index ERCP) were collected by retrospective chart review.

### Study outcomes

Patients were followed up for at least 12 months after the index ERCP. The 2 primary outcomes for this study were (1) diagnostic performance of the PB-FISH assay, defined as sensitivity, specificity, and positive and negative likelihood ratios and (2) rate and predictors of false-negative PB-FISH in biliary strictures. A priori subgroup analyses were performed in (1) participants with PSC-related strictures and (2) participants with biliary strictures unrelated to pancreatic ductal adenocarcinoma (PDAC), focusing on CCA versus benign biliary strictures.

### Statistical analysis

Discrete variables were summarized using frequencies and percentages. Sensitivity, specificity, disease prevalence, positive predictive value, negative predictive value, and accuracy are expressed as percentages. The 95% CIs for sensitivity, specificity, and accuracy were calculated using the “exact” Clopper–Pearson method. CIs for the likelihood ratios were computed using the “Log method.”

To assess the association between patient characteristics and the occurrence of false-negative PB-FISH results, logistic regression models were employed. Anatomical factors (such as stricture location and presence of PSC) and procedural factors (such as dilation prior to tissue sampling) were preselected for univariable analysis. The presence of a mass on cross-sectional imaging was excluded from the multivariable analysis as this was a variable used for clinically defining malignancy.

Multivariable models were constructed using a best subsets approach, which minimized the Akaike Information Criterion to identify the most suitable candidate models. The variables selected through this method showed similar results to those obtained using a cross-validated Lasso regression technique.

## RESULTS

**FIGURE 1:**
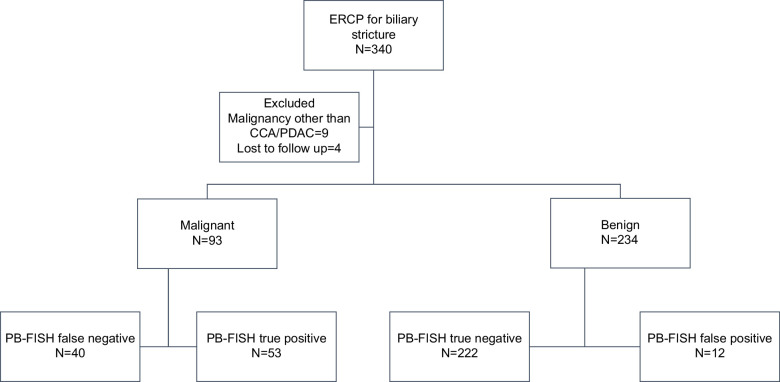
Study flowchart. Abbreviations: CCA, cholangiocarcinoma; ERCP, endoscopic retrograde cholangiopancreatography; PB-FISH, pancreatobiliary fluorescence in situ hybridization; PDAC, pancreatic ductal adenocarcinoma.

The final study cohort included 327 patients, including 130 (39.8%) with PSC. Thirteen patients were excluded, including 9 with malignancies other than CCA or PDAC and 4 due to lack of follow-up. Pancreatobiliary malignancy was diagnosed in 93 (28.5%) patients, 70 (75.2%) with CCA and 23 (24.8%) with PDAC (Figure 1). Malignancy was confirmed histopathologically in 75 (80.6%) patients, and the remainder had a diagnosis of malignancy based on established clinical criteria.

### Procedural characteristics

Procedural characteristics are reported in Table 1. A median of 1 (range 1–4) site was sampled. Amongst the 139 patients with >1 site of sampling, 100 (71.9%) of these had PSC. Common bile duct (CBD) alone was sampled in 107 (32.7%) cases, followed by perihilar sampling alone in 110 (33.6%) cases, and CBD plus perihilar sampling in 101 (30.9%) patients. Fifty-six (17.1%) of patients had a prior history of endoscopic biliary sampling. All patients had biliary brushing performed, while 216 (66.1%) had dilation performed as well. Dilation was performed prior to brushing in 180 (55.0%) cases. Cross-sectional imaging with CT/MRI within 30 days of the procedure was available in 260 (79.5%) patients, which showed a mass in 89 (34.2%) of these patients.

**TABLE 1:** Baseline characteristics, procedural details and outcomes of patients with biliary strictures stratified by a false-negative pancreatobiliary fluorescence in situ hybridization (PB-FISH) assay result

		PB-FISH results		
	All patients (N=327)	False-negative PB-FISH (N=40)	Others (N=287)	*p*
Mean age (SD), y	59.1 (16.6)	64.2 (16.4)	59.4 (16.5)	0.42
Female sex	136 (41.6%)	16 (40%)	120 (41.8%)	0.83
Race				0.11
White	306 (93.6%)	35 (87.5%)	271 (94.4%)	
African American	6 (1.8%)	0 (0.0%)	6 (2.1%)	
Asian	6 (1.8%)	2 (5.0%)	4 (1.2%)	
Others	9 (2.7%)	3 (7.5%)	6 (2.1%)	
Hispanic or Latino ethnicity	9 (2.7%)	2 (5.0%)	7(2.4%)	0.08
Primary sclerosing cholangitis	130 (39.8%)	8 (20.0%)	122 (42.5%)	<0.01
Mass on cross-sectional imaging	89 (34.2%)	30 (75.0%)	59 (26.8%)	<0.01
Prior ERCP-guided sampling	56 (17.1%)	3 (7.5%)	53 (18.5%)	0.03
Site of sampling				0.10
** **Hilar only	110 (33.6%)	18 (45.0%)	92 (32.1%)	
** **CBD with or without hilar sampling	217 (66.4)	22 (55.0)	195 (67.9)	
Total number of sampling sites	216 (66.1%)	25 (62.5%)	191 (66.6%)	0.02
** **1	188 (57.5%)	30 (75.0%)	158 (55.1%)	
** **2	51 (15.6%)	3 (7.5%)	48 (16.7%)	
** **3	43 (13.1%)	1 (2.5%)	42 (14.6%)	
** **>3	45 (13.7%)	6 (15.0%)	39 (13.5%)	
Dilation prior to brushing	180 (55.0%)	20 (50.0%)	160 (55.7%)	0.61
Malignancy status				
** **Benign	234 (81.5%)	0	234 (81.5%)	
** **Malignant	93 (28.4%)	40 (100%)	53 (18.5%)	
** **Pancreatic ductal adenocarcinoma	23 (7.0%)	16 (40.0%)	7 (2.4%)	
** **Cholangiocarcinoma	70 (21.4%)	24 (60%)	46 (16.0%)	

Continuous variables are presented as mean (SD) and categorical variables as n (%).

Abbreviations: CBD, common bile duct; ERCP, endoscopic retrograde cholangiopancreatography; PB-FISH, pancreatobiliary fluorescence in situ hybridization.

### Performance of BC and PB-FISH

**FIGURE 2:**
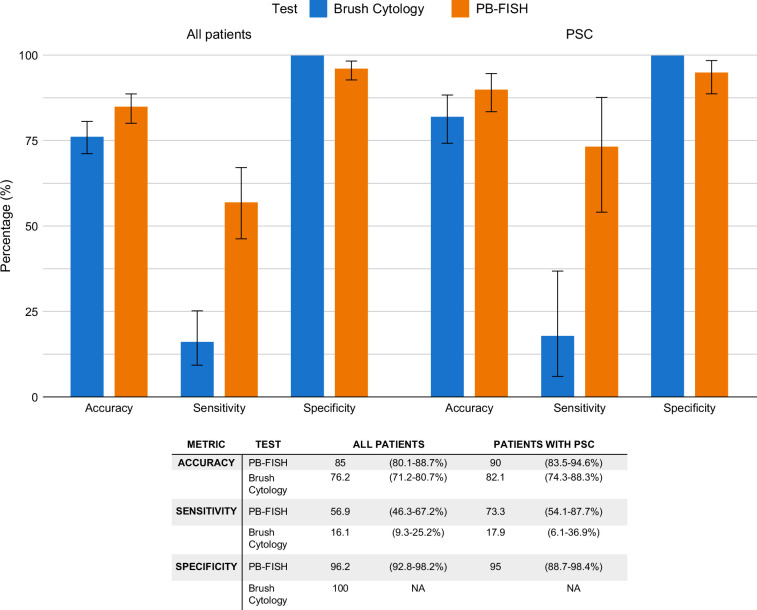
Diagnostic performance of PB-FISH for diagnosis of CCA or PDAC in biliary stricture stratified by all patients (blue) and patients with primary sclerosing cholangitis (orange). Abbreviations: CCA, cholangiocarcinoma; PB-FISH, pancreatobiliary fluorescence in situ hybridization; PDAC, pancreatic ductal adenocarcinoma; PSC, primary sclerosing cholangitis.

**FIGURE 3:**
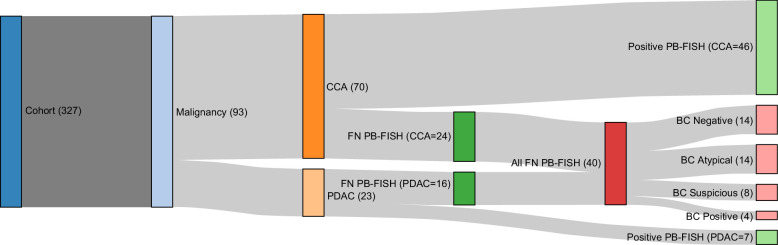
Sankey diagram illustrating patient flow from the total cohort to final diagnosis (CCA and PDAC), PB-FISH results, and corresponding BC findings. Abbreviations: BC, brush cytology; CCA, cholangiocarcinoma; PB-FISH, pancreatobiliary fluorescence in situ hybridization; PDAC, pancreatic ductal adenocarcinoma; PSC, primary sclerosing cholangitis.

Diagnostic characteristics of BC and PB-FISH are reported in Figure 2. A false-negative BC and PB-FISH were noted in 83.9% (n=78) and 43.1% (n=40) patients, respectively. PB-FISH was falsely negative in 69.6% of patients with PDAC (16/23), whereas 34.3% of patients with CCA had a falsely negative PB-FISH. A breakdown of PB-FISH results in these patients is shown in Table 2. Cytology results were reported as negative (14), atypical (14), suspicious (8), and positive (4) in the 40 individuals with false-negative PB-FISH (Figure 3). Of particular note, 3 of those 4 individuals with malignant cytology despite a negative PB-FISH result were subsequently diagnosed with CCA, while 1 had PDAC.

**TABLE 2:** PB-FISH results in patients with a false-negative PB-FISH

Patients with false-negative PB-FISH		
PB-FISH result	All patients (n=40)	Patients with primary sclerosing cholangitis only (n=8)
Negative	29	7
SLG	6	1
Homozygous 9p21 loss	2	0
SLG with homozygous 9p21 loss	3	0
Insufficient	2	0

Abbreviations: PB-FISH, pancreatobiliary fluorescence in situ hybridization; SLG, Single locus gain.

In the 24 participants with CCA that had a false-negative PB-FISH, the eventual cancer diagnosis was made by biopsy of a metastatic lesion in 11 cases (4 by EUS and 7 by percutaneous sampling of distant metastasis); 9 based on established clinico-radiological criteria based on AASLD guidelines. Among the 9 patients who underwent clinico-radiological diagnosis, 3 underwent surgery, and all were confirmed to have CCA on the surgical specimen. Finally, malignancy was diagnosed in 4 patients on intraductal sampling during the same ERCP (3 with BC and 1 with both positive BC and intraductal biopsy).

### Predictors of false-negative PB-FISH and BC

**FIGURE 4:**
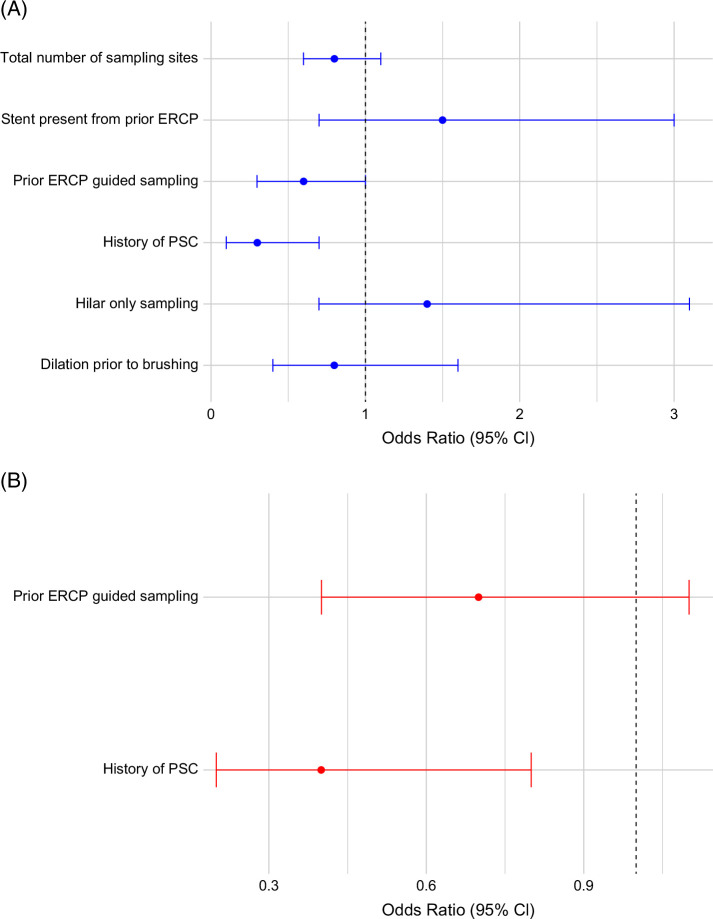
Univariable (A) and multivariable (B) analysis of factors associated with false-negative PB-FISH in all patients with biliary strictures. Abbreviations: ERCP, endoscopic retrograde cholangiopancreatography; PB-FISH, pancreatobiliary fluorescence in situ hybridization; PSC, primary sclerosing cholangitis.

Univariable and multivariable analyses of predictors of false-negative PB-FISH are reported in Figure 4. On multivariable analysis, history of PSC was associated with a lower risk of a false-negative PB-FISH test result (aOR=0.38, 95% CI=0.16–0.82, *p*=0.02) (Figure 4). The CA 19-9 levels between patients with false-negative PB-FISH were similar to those with a true positive PB-FISH (median 95 [IQR 25–287] IU/mL vs. 97.5 [IQR 35.5–279] IU/mL; *p*=0.91). The CA 19-9 level was not associated with a false-negative PB-FISH on univariable analysis (OR 1.0 [95% CI 0.99–1.00]).

Predictors of false-negative BC: Presence of a mass on imaging (aOR=9.5 [5.2–18.1], *p*<0.001) and presence of stent prior to sampling (aOR=2.3 [1.1–4.5], *p*=0.02) were both associated with false-negative BC.

### Subgroup analysis

#### Individuals with biliary strictures unrelated to PDAC (which included both CCA and benign etiologies)

**FIGURE 5:**
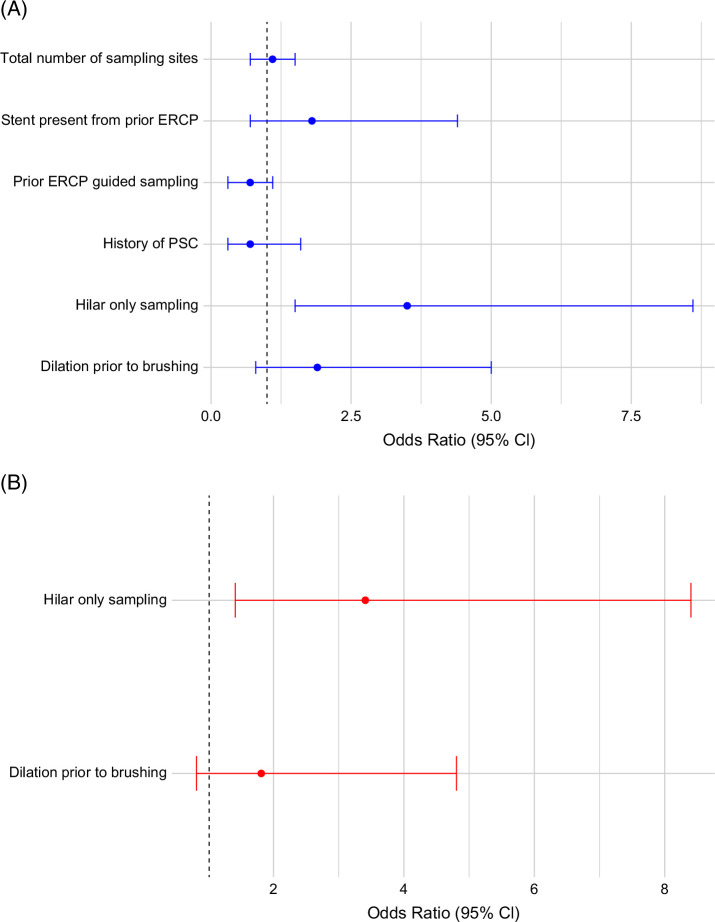
Univariable (A) and multivariable (B) analysis of factors associated with false-negative PB-FISH in patients with cholangiocarcinoma with a negative PB-FISH. Abbreviations: ERCP, endoscopic retrograde cholangiopancreatography; PB-FISH, pancreatobiliary fluorescence in situ hybridization; PSC, primary sclerosing cholangitis.

PB-FISH was falsely negative in 24 of the 70 patients with CCA (34.2%). On univariable analysis among patients with a negative PB-FISH, sampling of a hilar stricture was associated with a false-negative PB-FISH (Figure 5). On multivariable analysis, hilar sampling alone (aOR=3.2 [1.4–7.9], *p*=0.009) remained as the strongest predictor of false-negative PB-FISH (Figure 5B).

#### Individuals with PSC

In total, there were 130 patients with PSC. Diagnostic performance of BC and PB-FISH is reported in Table 2 and Figure 2. False-negative PB-FISH was noted in 8 (6.2%) patients, whereas false-negative BC was observed in 25 patients (19.2%). The PB-FISH findings of these patients are depicted in Table 2. Univariable analysis of factors predictive of false-negative PB-FISH suggested a trend toward higher likelihood of false-negative PB-FISH with a greater number of sampling sites (OR=1.76 [0.94–3.72], *p*=0.09) (Supplemental Table S1, http://links.lww.com/HC9/C48).

### Outcomes based on PB-FISH results

The time to cancer diagnoses was longer in those with a false-negative PB-FISH (median time 1.5 days [IQR=0–34.5]) in comparison with those with a true positive PB-FISH (median time 0 days [IQR 0]). However, the candidacy for curative resection was similar between the 2 groups. Amongst participants with a positive PB-FISH (n=53), 30 (56.6%) had resectable disease while 23 (43.4%) had unresectable disease at the time of initial presentation. On the other hand, in the 40 participants with a false-negative PB-FISH, 19 (47.5%) had resectable disease while 21 (52.5%) patients had unresectable disease. These differences were not statistically significant (*p*=0.38).

## DISCUSSION

PB-FISH has demonstrated improved diagnostic sensitivity for malignancy in biliary strictures over traditional BC. In this study, the sensitivity of PB-FISH was modest at 56.9% but demonstrated very high specificity at 96.2%. Factors associated with FISH performance characteristics have not been well elucidated. A history of PSC was associated with lower odds of having a false-negative PB-FISH, whereas a hilar stricture was associated with a significantly higher false-negative PB-FISH in those with CCA. Dilation of biliary strictures prior to brushing was not associated with a lower rate of false-negative PB-FISH.

False-negative FISH results are common in biliary strictures and are likely a combination of anatomical and procedural factors and the type of malignancy. This study attempted to further investigate each of these factors. A significantly higher proportion of patients with PDAC had a false-negative PB-FISH (69.6%) in comparison with those with CCA (34.3%). This stark difference is explained by the nature of biliary strictures in these 2 conditions. While CCA originates in the biliary epithelium, PDAC-related biliary strictures are more likely a result of external compression without any notable chromosomal abnormalities in most cases.^11^ The lack of chromosomal abnormalities consequently underestimates the sensitivity of PB-FISH in PDAC, and this should be factored in when evaluating a negative PB-FISH test in patients with a high clinical suspicion for PDAC. These findings align with current guidelines, which suggest single-session endoscopic ERCP ultrasound (EUS)-guided sampling for biliary strictures associated with a pancreatic mass.^2^ Additionally, our findings further illustrate the independent yet complementary diagnostic roles of cytology and PB-FISH. Four patients in this study had malignant cytology despite a negative PB-FISH. Such discordant findings are likely related to tumor heterogeneity, where cells either lack or express at lower levels the specific genetic alterations that the PB-FISH assay is designed to detect. Chromosomal aberrations that do not fall under the commonly targeted loci may contribute to a false-negative FISH, while cytology can still identify malignancy through morphological hallmarks (eg, nuclear atypia, irregular chromatin, and high nuclear-to-cytoplasmic ratio). Given these considerations, this data reinforces the importance of a multimodal approach, combining cytology and PB-FISH, to maximize diagnostic accuracy in evaluating biliary strictures.

Dilation of strictures to increase tumor exfoliation has been postulated to improve the diagnostic yield of BC. In this study, dilation of stricture prior to brushing was not associated with lower odds of false-negative PB-FISH (Figure 4). Our findings align with those observed in 2 prior retrospective studies where dilation of strictures did not improve the diagnostic yield of BC.^12^^–^^14^ However, in patients with CCA, on multivariable analysis, there was a trend toward a higher rate of false-negative PB-FISH in patients with stricture dilation prior to brushing in the present study. Disruption of normal biliary epithelium and perihilar location likely accounted for the higher rate of false-negative PB-FISH in patients with CCA. CCA usually spreads subepithelially, and thus adequate sampling in perihilar strictures can be challenging, potentially contributing to higher odds of false-negative PB-FISH.^15^ Several other procedural techniques have been tried to optimize sampling, including larger or angled brushes, repeat brushings or higher number of passes, scraping devices and use of stent retrievers with variable impact on yield for BC, but their impact on PB-FISH has not yet been evaluated.^16^^–^^20^ Future randomized studies to evaluate different techniques and their order of use are needed to define the optimal sampling strategy and enhance sensitivity.

Location of biliary strictures can also impact the performance of PB-FISH. Indeed, patients with CCA that had only perihilar strictures had 3-fold higher odds of a false-negative PB-FISH, which is likely multifactorial. First, ERCP-guided sampling in the hilar region can be technically challenging due to narrowed ducts and the difficulty of maneuvering an endoscopic brush or forceps. This limitation is consistent with prior studies demonstrating lower sensitivity of BC and forceps biopsy for perihilar strictures. Second, hilar CCA often exhibits a submucosal or sclerotic growth pattern and induces a dense desmoplastic (fibrotic) reaction, which can reduce the number of tumor cells available for sampling. Additionally, anatomical complexity at the hepatic duct confluence makes it challenging to access the stricture adequately, and these tumors frequently have limited luminal involvement, thus shedding fewer cells into the duct. In this study, Patients with only hilar strictures were more likely to have de-novo CCA in comparison to those with CBD or CBD and hilar strictures (78.8% vs. 37.8%, *p*<0.01) and more likely to have a mass on imaging (84.8% vs. 61.8%, *p*=0.03). Although we could not ascertain the specific pattern of tumor spread, mass-forming perihilar CCA has limited intraductal spread and often compresses the duct, making intraductal sampling more difficult and potentially leading to false-negative findings. These results suggest that in patients with hilar strictures without CBD involvement, a negative PB-FISH alone should not be the basis to rule out CCA. These individuals should be closely monitored with the potential need for repeat intervention to prevent a further delay in the diagnosis of malignancy. In the same session, EUS and ERCP can be considered, as it has been shown to have higher diagnostic sensitivity in these scenarios.^21^ However, it must be noted that EUS-guided sampling of primary CCA should not be performed when liver transplantation is being considered. Transperitoneal sampling of perihilar CCA is associated with a higher rate of peritoneal metastasis and is considered a contraindication for liver transplantation.^22^

A history of PSC was associated with lower odds of having false-negative PB-FISH in this study. PB-FISH assay was designed to detect PB malignancies, and the high sensitivity (73.3%, 95% CI [55.6%–85.8%]) in this study supports the use of the optimized PB-FISH assay in PSC patients for CCA detection. The lower odds of false-negative PB-FISH are likely related to multiple factors, including the fact that the PB-FISH assay was developed for the detection of pancreatobiliary malignancy using probes to chromosomal regions aberrant in these cancers, and secondly, the broader field defect in PSC. Cancer in PSC arises in the background of widespread chronic inflammation throughout the biliary tree, and the PB-FISH assay can detect not only adenocarcinoma but also high-grade dysplasia, which harbors similar cytogenetic abnormalities. Additionally, due to the higher lifetime risk of CCA in PSC, there is a lower threshold to obtain biliary brushings and likely more sampling, further improving sensitivity. Among those with a false-positive PB-FISH, polysomy was also noted only on one site and on close follow-up, serial polysomy was not detected. In patients with PSC, a negative PB-FISH should be interpreted with caution when a mass is seen on imaging. In contrast, unifocal polysomy without an associated mass in PSC patients could be followed up with close monitoring in the appropriate clinical setting, as this may represent low-grade dysplasia rather than high-grade dysplasia or malignancy. Current guidelines recommend short-interval repeat ERCP if polysomy is present without an associated mass.^9^

This study has some limitations. It was a prospective nonrandomized design where different techniques, such as biliary dilation, order of brushing, and dilation, were performed at the discretion of the performing endoscopist. However, sampling was performed by multiple experienced endoscopists, thus reducing provider bias. Secondly, cross-sectional imaging was not available in all patients immediately prior to undergoing endoscopic evaluation. The overall prevalence of malignancy in this cohort of patients with biliary strictures was lower than in prior studies.^2^ This is reflective of a significant population of PSC patients undergoing regular endoscopic evaluation and tissue sampling at our institution. Although we studied the impact of prior ERCP-guided sampling, the specific details of prior techniques were not recorded. Consequently, the impact of prior interventions (eg, dilation) may have on sampling yields in the subsequent procedures could not be studied.

In conclusion, although significantly more sensitive than BC for detecting malignancy, a false-negative PB-FISH is common after ERCP-guided sampling. Hilar strictures were associated with a higher risk of false-negative PB-FISH in patients with perihilar CCA. Dilation of strictures prior to brushings did not increase sampling yield for PB-FISH. In patients with a mass on imaging, a negative PB-FISH should not be the basis for ruling out PB malignancy and further workup and close follow-up are recommended. The results of this study can guide the interpretation of a negative PB-FISH in patients with biliary strictures with and without PSC. Guarantor of the article: Vinay Chandrasekhara, MD.

## Supplementary Material
