# Supplementary material for: The incidence and predictors of false-negative pancreatobiliary fluorescence in situ hybridization (PB-FISH) in biliary strictures: A prospective study
Source: Hepatol Commun. 2025 Jul 14;9(8):e0751. doi: 10.1097/HC9.0000000000000751 (PMC12262981; doi:10.1097/HC9.0000000000000751)
Supplement: Supplementary file 1 [file hc9-9-e0751-s001.pdf]

Supplementary table

Supplementary table 1: Univariable and multivariable analysis of factors associated with false negative pancreatobiliary fluorescence in situ hybridization (PB-FISH) in patients with primary sclerosing cholangitis only.

	Univariable analysis		
	**Univariable odds**	**p.value**	**N miss**
**Hilar only sampling**	0.63 (0.09-2.89)	0.58	0
**Total number of sampling sites**	1.76 (0.94-3.72)	0.09	0
**Dilation prior to brushing**	1.21 (0.26-8.51)	0.82	0
**Prior ERCP guided sampling**	0.6 (0.14-1.27)	0.32	0
**Stent present from prior ERCP**	1.1 (0.06-6.88)	0.93	0

Abbreviations: ERCP, endoscopic retrograde cholangiopancreatography; PSC, primary sclerosing cholangitis.
